# 

**Published:** 1886-08

**Authors:** 


### CONTENTS.

COMMUNICATIONS.

Physiology and Pathology of First Dentition .......... 373
Address on Dental and Oral Surgery .......... 377
Syphilis in its Relation to Dental and Oral Surgery .......... 388
Mechanical Dentistry .......... 401

PROCEEDINGS.

Georgia State Dental Society .......... 404

SELECTIONS.

The Wear and Tear of Modern Life .......... 415
Atrophic Progressive Myopathy .......... 418
Dental Erosion in Scrofula .......... 420
Death of Dr. C. Robin .......... 420

SALMAGUNDI .......... 421

